# Familial Autism Spectrum Disorder : A clinical study from South Tunisia

**DOI:** 10.1192/j.eurpsy.2023.844

**Published:** 2023-07-19

**Authors:** I. Boujelben, M. Chaabane, I. Ben ayed, D. Ben Touhemi, N. Gharbi, M. Guirat, I. HajKacem, H. Ayadi, H. Kamoun, Y. Moalla

**Affiliations:** 1 Department of Medical Genetic; 2 Department of Child Psychiatry, Hedi Chaker Hospital, Sfax, Tunisia

## Abstract

**Introduction:**

Autism Spectrum Disorder (ASD) is a multifactorial neurodevelopmental disorder, with both contribution of genetic and non-genetic factors. A collaboration of *de novo* mutations and prenatal with postnatal environmental factors are likely to play a role. ASD can be syndromic or non-syndromic. The etiology of non-syndromic ASD is still relatively undefined due to its genetic heterogeneity. Contrary to non-syndromic ASD, syndromic ASD is often associated with chromosomal abnormalities or monogenic alterations. Familial cases of ASD support the strong genetic component of ASD.

**Objectives:**

To collect clinical arguments supporting a genetic cause of autism spectrum disorder.

**Methods:**

We present a clinical study of familial cases of ASD. The families were recruited as part of a collaborative project between the department of Medical Genetic and the department of Child Psychiatry in Hedi Chaker Hospital, Sfax, Tunisia. The clinical and paraclinical data were collected retrospectively.

**Results:**

Our study included 11 unrelated families from southern Tunisia, each with two ASD children, among them four couples of twins. Most families (80%) are consanguineous belonging to a middle socioeconomic class. None of the parents suffered from psychiatric disorder and a familial history of autism was reported in one family. Perinatal history, including advanced maternal or paternal age, fetal suffering and/or gestational problems, was found in 35% of cases.

The average age was 9.89 ± 3.787 (3 to 17 years) with a balanced sex-ratio.

ASD was syndromic in seven out of 11 families: facial dysmorphism in half of cases (6/11 families) and/or another comorbidity in 25% of cases (celiac disease, congenital heart disease or idiopathic hydrocephaly). ASD was associated with other(s) neurodevelopmental disorder(s) in all children. Most of cases (14/22) had delayed psychomotor development and all of them had intellectual disability with various degrees. Epilepsy was identified in three cases belonging to unrelated families. Other behavioral problem was identified in 65% of cases.

When the autism spectrum disorder is syndromic and/or associated with other(s) neurodevelopmental(s) disorder(s), this points more towards a genetic origin.

**Conclusions:**

Our study highlights the interest of clinical investigations to determine genetic risk factors of ASD. The identification of a genetic cause in familial cases would contribute not only to better understand the pathological processes of ASD but also to provide an appropriate genetic counseling.

**Disclosure of Interest:**

None Declared

